# Ten-Year Trends in First- and Second-Line *Helicobacter pylori* Eradication Rates at a Regional Hospital in Japan

**DOI:** 10.14789/ejmj.JMJ26-0004-OA

**Published:** 2026-04-22

**Authors:** YUJI SHIMADA, MARIKO HOJO, HIROKI NAGO, MASAHIRO YAMAGUCHI, YOKO KATO, RIHWA OM, YUICHIRO TERAI, YUJI KITA, YUJI IKEDA, SHO SATO, AYATO MURATA, SHUNSUKE SATO, AKIHITO NAGAHARA, TAKUYA GENDA

**Affiliations:** 1 Department of Gastroenterology and Hepatology, Juntendo University Shizuoka Hospital, Shizuoka, Japan; 2 Department of Gastroenterology and Hepatology, Juntendo University Shizuoka Hospital, Shizuoka, Japan; 2 Department of Gastroenterology and Hepatology, Juntendo University School of Medicine, Tokyo, Japan; 2 Department of Gastroenterology and Hepatology, Juntendo University School of Medicine, Tokyo, Japan

**Keywords:** *Helicobacter pylori*, eradication, vonoprazan, clarithromycin resistance, real-world data

## Abstract

**Objectives:**

*Helicobacter pylori* (*H. pylori*) infection is a major risk factor for gastric cancer, and successful eradication reduces long-term cancer incidence. Since national insurance coverage expanded in Japan in 2013, to include *H. pylori*-positive gastritis, eradication therapy has become widely accessible; however, outcomes may vary with antimicrobial resistance, evolving treatment regimens, and regional prescribing patterns. We investigated 10-year trends in eradication efficacy at a regional hospital in Japan.

**Methods:**

This retrospective observational study included all patients who underwent first- or second-line *H. pylori* eradication therapy in our department between 2015 and 2024. Clinical characteristics, treatment regimens, and post-treatment test results were extracted from medical records. Annual success rates were assessed by intention-to-treat (ITT) and per-protocol (PP) analyses and evaluated using binomial generalized linear models, with a descriptive linear regression. Regimen selection and institutional clarithromycin utilization were also assessed for temporal trends.

**Results:**

Among 1,246 patients (1,126 first-line; 120 second-line), first-line eradication rates were stable in ITT analyses, with a modest upward trend in PP analyses. Second-line eradication rates remained consistently high without a significant temporal trend. Vonoprazan-based regimens became predominant, while institutional clarithromycin utilization declined.

**Conclusions:**

Over the past decade, first-line *H. pylori* eradication efficacy remained stable in ITT analyses with modest improvement in PP analyses, while second-line therapy maintained high performance. The widespread establishment of vonoprazan-based regimens likely ensured stable efficacy, with a plausible additional contribution from declining clarithromycin use at the regional level. These findings provide insight into long-term, real-world eradication trends and may help inform treatment strategies in similar clinical settings.

## Introduction

*Helicobacter pylori* (*H. pylori*) infection is a well- established risk factor for gastric cancer, and eradication therapy has been shown to significantly reduce long-term cancer incidence^1-4)^. Following the expansion of national insurance coverage for eradication therapy in Japan in 2013, treatment has become widely accessible across all age groups, contributing to population-level gastric cancer prevention^5)^. Multiple long-term studies, including randomized trials and cohort analyses, have shown that successful *H. pylori* eradication markedly lowers the risk of developing gastric cancer^1-4)^.

Real-world eradication outcomes, however, may change over time due to factors such as shifts in antimicrobial resistance^6, 7)^, particularly clarithromycin resistance, evolving treatment regimens including the adoption of potassium-competitive acid blockers (P-CABs)^8)^, variations in patient adherence^9)^, and regional prescribing patterns that can influence macrolide resistance levels^6, 10^^-^^12)^. Understanding these influences is essential for interpreting temporal trends in eradication success.

The Izu region of Shizuoka Prefecture, where our hospital is located, has a notably aging population compared with major urban areas in Japan^13)^. This demographic context may affect local patterns of treatment demand and antimicrobial resistance, yet long-term real-world data from such communities remain limited.

Given this background, clarification of how eradication outcomes have shifted over time in this regional setting has important implications for clinical practice and public health. Therefore, the aim of this study was to assess 10-year trends in first- and second-line *H. pylori* eradication success rates among patients treated exclusively in our gastroenterology department between 2015 and 2024.

## Materials and Methods

### Study design and setting

This was a retrospective observational study conducted at a regional hospital located in Shizuoka Prefecture, Japan. The study period spanned ten years, from January 2015 to December 2024.

### Study population

We included all patients who underwent first- or second-line *H. pylori* eradication therapy and were managed exclusively by our department during the study period. Patients who received eradication therapy in other departments were not included. Clinical data extracted from medical records included age, sex, eradication regimen, and results of post-treatment testing.

### Eradication regimens

First-line eradication therapy typically consisted of PPI- or P-CAB-based triple therapy using amoxicillin and clarithromycin. The standard regimen was administered for 7 days and comprised amoxicillin (1,500 mg/day) plus clarithromycin (400 or 800 mg/day), combined with an acid suppressant (vonoprazan 40 mg/day; TAKECAB^®^, Takeda Pharmaceutical Co., Ltd., Tokyo, Japan, or a PPI: omeprazole 40 mg/day, lansoprazole 60 mg/day, rabeprazole 20 mg/day, or esomeprazole 40 mg/day). Acid suppressants and antibiotics were administered as branded or generic products available at our hospital during the study period. In cases of penicillin allergy or other contraindications, alternative non-standard regimens were used. For patients with penicillin allergy, first-line eradication therapy generally consisted of an acid suppressant plus clarithromycin and metronidazole for 7 days (clarithromycin 400 or 800 mg/day; metronidazole 500 mg/day). For patients with clarithromycin allergy, an acid suppressant plus amoxicillin and metronidazole for 7 days was used (amoxicillin 1,500 mg/day; metronidazole 500 mg/day). These patients were included in the first-line cohort.

Second-line therapy consisted of PPI- or P-CAB-based triple therapy using amoxicillin and metronidazole. The standard regimen was administered for 7 days and comprised amoxicillin (1,500 mg/day) plus metronidazole (500 mg/day), combined with the same PPI- or P-CAB-based acid suppression as described above. Metronidazole was administered as a branded or generic product available at our hospital during the study period. Regimen choice varied by year according to national recommendations and institutional prescribing practices.

### Confirmation of eradication

Eradication was confirmed using either the ^13^C- urea breath test or a stool antigen assay. The ^13^C- urea breath test was performed using UBIT^®^ (Otsuka Pharmaceutical Co., Tokyo, Japan). A Δ^13^CO_2_ value < 2.5‰ was considered negative, while values ≥ 2.5‰ and < 5.0‰ were regarded as a gray zone, in which case the test was repeated three months later; only patients whose repeat value was ≤2.5‰ were classified as negative (successful eradication). Stool antigen testing was performed using a monoclonal antibody-based enzyme immunoassay (Testmate *H. pylori* Antigen EIA^®^, Canon Medical Diagnostics, Tokyo, Japan). Positivity and negativity were defined according to the manufacturer’s criteria: optical density (OD) < 0.140 (single-wavelength, 450 nm) or < 0.100 (dual-wavelength, 450/ 630 nm) was considered negative.

### Outcome measures

The primary outcomes were the annual first- and second-line eradication success rates over the 10-year study period. Both intention-to-treat (ITT) and per-protocol (PP) analyses were performed. For PP analysis, patients with ≥ 80% medication adherence were considered eligible. In addition, the annual proportions of vonoprazan-based and PPI- based regimens were extracted from each yearly dataset to evaluate temporal changes in regimen selection patterns.

### Clarithromycin utilization data

Institutional clarithromycin utilization data were obtained from internal pharmacy purchasing records. These data were anonymized and analyzed in aggregated form, and were used to explore temporal patterns in macrolide use that might relate to regional clarithromycin resistance. These data were analyzed as contextual information to aid interpretation of temporal changes in eradication outcomes, rather than as a primary study outcome.

### Statistical analysis

Statistical analyses were performed using both model-based and descriptive approaches. Annual eradication outcomes were evaluated using generalized linear models (GLMs) with a binomial distribution and a logit link, treating calendar year as a continuous predictor. Descriptive trends were additionally illustrated using simple linear regression. Temporal trends in the annual proportion of vonoprazan-based versus PPI-based regimens were assessed using the Cochran-Armitage trend test. Clarithromycin prescription counts were analyzed as count data using Poisson regression models with calendar year as the explanatory variable; negative binomial regression was applied when overdispersion was present. Linear regression of annual prescription counts was also performed as a descriptive supplement. All statistical tests were two-sided, with p values < 0.05 considered statistically significant.

### Ethical considerations

This study was conducted in accordance with the Declaration of Helsinki. The protocol was approved by the Juntendo University Medical Research Ethics Committee (Approval No. C22- 0175). Although the approval was obtained under a multicenter retrospective study framework, the present analysis was conducted using only anonymized data from Juntendo University Shizuoka Hospital. Because only retrospective, anonymized data were used, the requirement for written informed consent was waived in accordance with institutional regulations.

## Results

### Patient characteristics

**Supplementary Figure S1:**
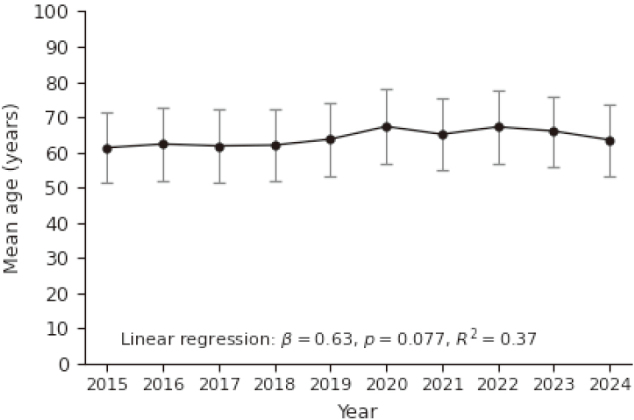
Annual mean age of patients receiving first-line eradication therapy Yearly mean patient age is shown with 95% confidence intervals. No significant temporal change in age distribution was observed during the study period.

A total of 1,246 patients underwent *H. pylori* eradication therapy at our department between 2015 and 2024. Among these, 1,126 received first-line eradication therapy and 120 underwent second- line therapy. The mean age of patients receiving first-line therapy was 67.4 years (IQR, 55-74), and 51.0% were male. In the second-line group, the mean age was 66.1 years (IQR, 52-73), and 44.2% were male. The annual mean age of patients who received first-line eradication therapy remained largely stable throughout the study period, as shown in Supplementary Figure S1.

### Temporal trends in case volume

**Supplementary Figure S2:**
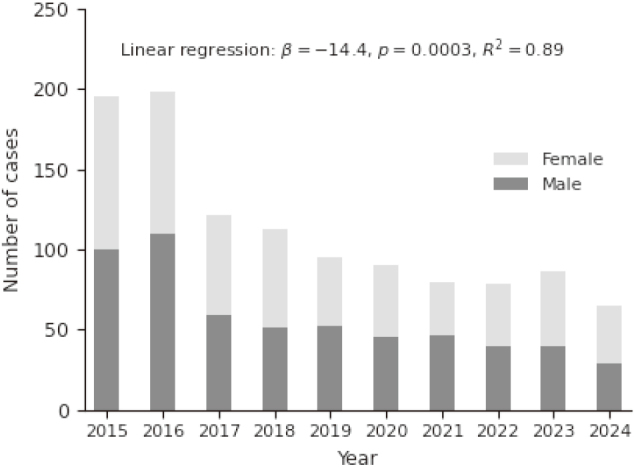
Annual number of patients undergoing first-line eradication therapy Yearly case volume demonstrated a significant declining trend from 2015 to 2024. Linear regression analysis indicates a progressive reduction in the number of patients requiring first-line eradication therapy, consistent with saturation of eradication uptake in the region.

The annual number of patients undergoing first-line eradication therapy showed a gradual decline from 2015 to 2024 (Supplementary Figure S2). Linear regression analysis demonstrated a statistically significant downward trend (β = −14.4 cases per year, p = 0.0003, R^2^ = 0.89). In contrast, the annual number of second-line eradication cases remained small throughout the study period, with no clear increasing or decreasing pattern observed over time.

### First-line eradication outcomes

**Figure 1:**
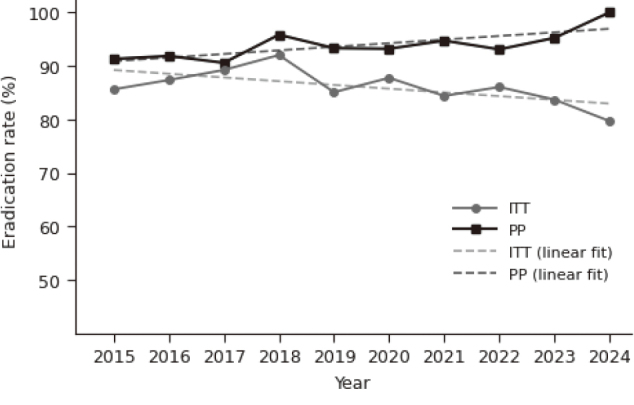
First-line eradication rates (ITT and PP) from 2015 to 2024

For first-line eradication therapy, the overall eradication success rate was 86.5% in the ITT analysis (967/1,118) and 92.8% in the PP analysis (633/ 682). As shown in Figure 1, PP-based eradication efficacy showed a gradual upward pattern over the 10-year period, whereas the ITT curve exhibited only limited annual fluctuation and remained largely stable throughout the decade. Binomial GLM identified no significant linear temporal trend in ITT- based outcomes (p = 0.153), while PP-based outcomes showed a modest but non-significant upward trend (p = 0.0986). Descriptive linear regression yielded consistent directional patterns.

### Second-line eradication outcomes

**Figure 2:**
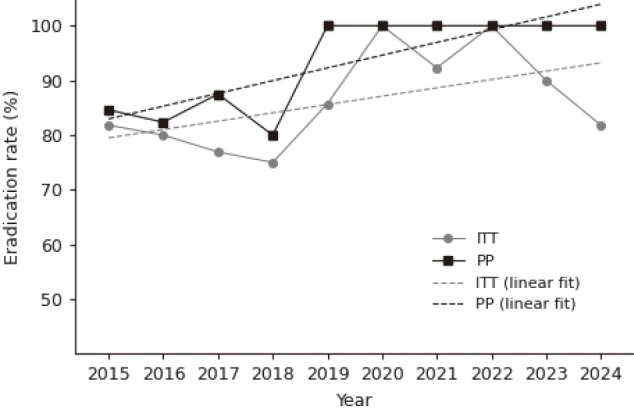
Second-line eradication rates (ITT and PP) from 2015 to 2024

For second-line therapy, the overall eradication success rate was 85.0% in the ITT analysis (102/ 120) and 90.3% in the PP analysis (11/13). Annual eradication rates showed modest variability before 2020 but became consistently high thereafter, with no apparent decline in subsequent years (Figure 2). Binomial GLM demonstrated no significant linear temporal trend for ITT-based outcomes (p = 0.2157), while PP-based outcomes showed a borderline positive trend (p = 0.0756). Descriptive linear regression results were in agreement with these observations.

### Regimen selection trends

**Figure 3:**
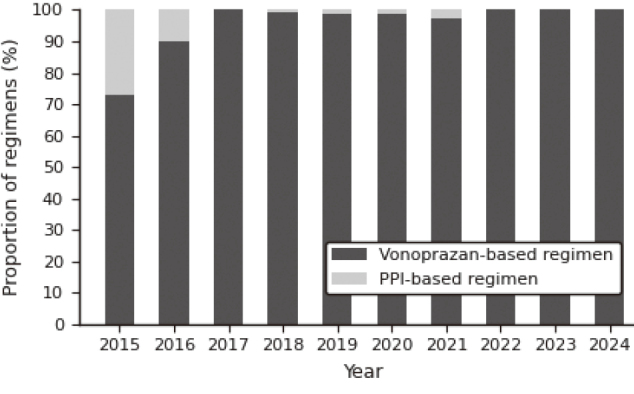
Annual proportion of vonoprazan- vs PPI-based regimens (2015-2024)

The proportion of vonoprazan-based regimens increased substantially over the study period, becoming the predominant first-line regimen after 2016. A significant increasing trend was confirmed by the Cochran-Armitage test (Z = 9.31, p < 0.001) (Figure 3).

### Clarithromycin utilization

**Figure 4:**
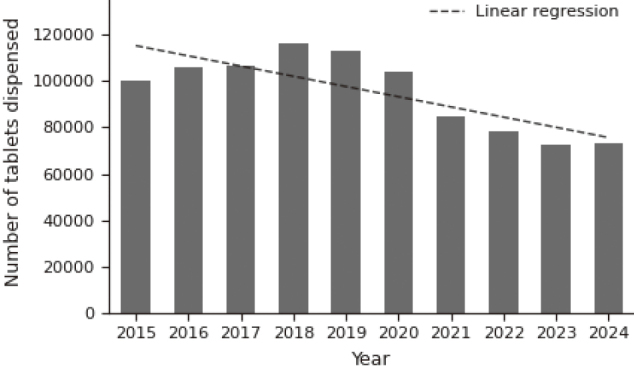
Annual clarithromycin use at our hospital

Institutional clarithromycin utilization steadily decreased from 2015 to 2024 (Figure 4). Poisson regression confirmed a significant temporal decline in prescription counts over the study period (β = −0.046, p < 0.001), corresponding to an average annual reduction of approximately 4.5%. A linear regression line is shown in Figure 4 for descriptive visualization, whereas statistical inference was based on Poisson regression.

## Discussion

This study retrospectively evaluated *H. pylori* eradication outcomes over a 10-year period (2015-2024) at our department. Overall, first-line eradication rates showed a modest improvement in PP analyses, whereas ITT-based outcomes remained largely unchanged over time. In contrast, second-line eradication rates remained consistently high, with only limited year-to-year variability.

As expected in the era of widespread P-CAB use, first-line eradication rates were generally favorable. PP-based eradication efficacy demonstrated a modest upward tendency, whereas ITT- based outcomes remained largely unchanged across the decade. Although the magnitude of improvement was small, the persistence of this upward trend—even after P-CAB-based regimens had become standard—suggested that factors beyond acid suppression might be contributing.

Several factors, including smoking, younger age, obesity, and delayed gastric emptying, have been associated with eradication failure^9, 14)^. However, clarithromycin (CAM) resistance remains the most important determinant of first-line treatment failure^9, 14, 15)^. Because vonoprazan maintains high eradication efficacy even against CAM-resistant strains^8, 14, 16)^, the widespread adoption of P-CAB-based regimens likely contributed to maintaining high eradication efficacy over time, consistent with current consensus statements emphasizing robust and sustained acid suppression^14, 17)^.

One hypothesis arose from long-standing evidence that antimicrobial exposure is associated with the development of clarithromycin resistance, as demonstrated by Meyer et al.^15)^, while regional macrolide consumption has been shown to correlate with resistance prevalence at the population level^10, 11)^. Because CAM resistance is a major driver of eradication failure^9, 11, 14, 15)^, even modest shifts in macrolide exposure could influence eradication outcomes at the population level. Motivated by this possibility, we examined institutional CAM prescribing patterns and observed a steady decline over the study period^12, 18)^. This finding led us to consider whether reduced macrolide exposure might have contributed, at least in part, to the gradual improvement in first-line eradication efficacy.

However, the relationship between antibiotic consumption and local resistance dynamics is not straightforward. Several surveillance studies have shown that CAM resistance does not necessarily decline rapidly even when macrolide use decreases, implying that resistance may persist once established^6, 19-22)^. At the population level, however, multiple ecological studies have consistently reported a positive association between macrolide consumption and resistance prevalence^10, 11)^. Thus, although current evidence is mixed, it remains biologically plausible that sustained reductions in CAM exposure could, over time, gradually create an environment less favorable for the persistence of clarithromycin- resistant strains. As nationwide macrolide consumption continues to fall in Japan^12, 23)^, the true impact of this trend may become clearer in the coming years.

The divergence between ITT and PP-based outcomes in our cohort also requires careful interpretation. A key explanation for this discrepancy is loss to follow-up after eradication therapy. A substantial number of patients did not return for post-treatment testing and were therefore classified as failures in the ITT analysis. This proportion increased during the COVID-19 pandemic, when many patients deferred outpatient visits^24)^. As a result, PP-based outcomes more accurately reflected the intrinsic performance of eradication regimens, whereas ITT-based outcomes were disproportionately affected by incomplete follow-up.

Second-line eradication therapy consistently demonstrated high efficacy throughout the study period. In the early phase of the study, a certain proportion of patients received PPI-based regimens; however, eradication efficacy was not substantially reduced during this period. Previous studies have reported that metronidazole-containing triple therapy achieves similarly high eradication rates with both PPI- and P-CAB-based regimens^9, 14)^, which may partly explain the stable second-line outcomes observed in this study.

This study has several limitations. First, it was a single-center retrospective analysis, and treatment patterns may differ in other settings. Second, CAM resistance was not routinely assessed, preventing direct evaluation of resistance dynamics. Third, the number of second-line cases was relatively small, which may have reduced statistical power for trend evaluation.

In summary, this study evaluated 10-year trends in first-line and second-line *H. pylori* eradication outcomes in a regional clinical setting. First-line eradication efficacy was generally favorable and demonstrated a modest upward tendency in PP analyses over time. These favorable first-line outcomes likely reflect the stable establishment of P-CAB-based therapy as the primary contributor. Additionally, at the regional level, declining clarithromycin use may have plausibly played a supportive role, possibly through favorable shifts in regional resistance patterns. In contrast, second-line eradication therapy consistently achieved high efficacy throughout the study period, with little temporal variation.

## Data availability

The datasets generated and/or analyzed during the current study are not publicly available due to ethical and privacy restrictions. They are available from the corresponding author on reasonable request and with appropriate institutional approvals.

## Author contributions

YS and AN conceived and designed the study. YS was primarily responsible for data collection. YS performed the statistical analyses and drafted the manuscript. MH and AN contributed to interpretation of the results. All authors read and approved the final manuscript.

## Conflicts of interest statement

The authors declare that there are no conflicts of interest.

## Supplementary Material
